# Developing and validating patient safety sessions in Japanese medical education: a modified Delphi study

**DOI:** 10.5116/ijme.69f0.78c6

**Published:** 2026-05-11

**Authors:** Ikuo Shimizu, Mariko Morishita, Misako Katori, Cocoro Hirai, Yumi Matsumura, Kazumi Tanaka

**Affiliations:** 1 Department of Medical Education, Chiba University Graduate School of Medicine, Japan; 2 Department of Patient Safety, Kyoto University Hospital, Japan; 3 Medical Safety Management Center, the University of Tokyo Hospital, Japan; 4 Department of Medical Safety Management, University of Tsukuba Hospital, Japan; 5 Department of Healthcare Quality and Safety, Gunma University Graduate School of Medicine, Japan

**Keywords:** Competency-based medical education, patient safety, undergraduate education, Delphi technique

## Abstract

**Objectives:**

This study aimed to (1) develop and validate educationally sound and feasible model session plans for patient safety learning in Japanese undergraduate medical education and (2) identify barriers to their implementation in educational settings.

**Methods:**

A convergent mixed-methods design was embedded within a modified Delphi study. Four session plans based on Merrill’s First Principles of Instruction were evaluated by 45 patient safety specialists and faculty purposively sampled from Japanese national university hospitals. Participants assessed plans using a 14-item Context–Input–Process–Product checklist. Items were rated on a four-point scale; consensus was defined as a mean score ≥ 3.5 and a standard deviation < 1.0. Simultaneously, open-ended comments on implementation challenges were analyzed using directed content analysis based on Steinert’s framework for educational barriers. Quantitative and qualitative data were integrated using a joint display to derive meta-inferences.

**Results:**

All four sessions met consensus criteria in the first round (n = 36; response rate = 80%). Topics covered human error, incident reporting, root cause analysis, and conflict management. Analysis identified 13 subthemes across five domains—teacher, student, knowledge, attitude, and system. Key barriers included limited faculty facilitation experience, insufficient linkage to safety practice, and institutional constraints such as lack of formal educational roles for safety specialists.

**Conclusions:**

The validated sessions demonstrated strong feasibility. However, content readiness alone does not guarantee successful adoption. Addressing identified barriers through targeted faculty development focusing on facilitation skills and organizational alignment is necessary to achieve sustainable implementation of patient safety education in undergraduate programs.

## Introduction

Over the past two decades, medical education has undergone a global transformation toward a competency-based approach, aligning curricula with the abilities required for safe and effective clinical practice.^1^ In Japan, the Model Core Curriculum for Medical Education was introduced to support this paradigm shift,^2^ and its 2022 revision emphasizes the overarching goal of “providing safe and high-quality healthcare.”^3^ Within this framework, patient safety learning is no longer viewed as a discrete subject but as a crucial cross-cutting domain that should be systematically integrated into all stages of undergraduate education. The World Health Organization (WHO) also underscores the necessity for systematic patient safety learning at the undergraduate level to enable future healthcare professionals to deliver safe and effective care.^4^ Particular emphasis is placed on cultivating safety culture, encompassing reporting and learning, early in the educational trajectory, with the aim of fostering student attitudes and practices that support the enhancement of healthcare quality and safety.^5^ The introduction of the longitudinal competency framework overarching undergraduate and residency curricula has further reinforced the continuous quality improvement of care.

Despite this global and national emphasis, the implementation of patient safety education for undergraduates remains limited in Japan. Structural barriers include a shortage of educators trained in patient safety, a lack of standardized instructional models, and insufficient linkage between safety education and clinical practice.^6^^,^^7^ A national survey reported that only around 30% of Japanese medical schools include certified patient safety specialists in their undergraduate curriculum planning,^7^ suggesting that most safety instruction is still delivered by faculty without formal training in safety science. In addition, undergraduate learning opportunities remain largely lecture-based, with few interactive or experiential activities.^8^ However, these barriers are diverse and multi-faceted, spanning individual, interpersonal, and organizational levels. To develop effective dissemination strategies, a systematic understanding of implementation challenges across multiple domains is essential.

In this study, several key terms are operationally defined. “Educationally valid” refers to session designs that are grounded in established educational theory and aligned with learning outcomes specified in the Model Core Curriculum. “Model sessions” denote transferable, evidence-informed lesson plans that can serve as templates for instructors seeking to implement or adapt patient safety learning activities in their institutions. These sessions are mapped to the core patient safety competencies outlined in the Japanese curriculum, including quality improvement, safety management systems, and practical safety practices.^2^

From a theoretical perspective, the limited translation of patient safety principles into undergraduate education can be viewed as a gap in applying funda-mental learning principles. According to Merrill’s First Principles of Instruction, effective learning occurs when learners are engaged in authentic problem-solving and can integrate new knowledge into their prior experiences.^9^ This study therefore approached the implementation gap in patient safety education as an instructional design problem—where traditional lecture-based approaches fail to activate prior knowledge or provide opportunities for application and integration. Merrill's First Principles of Instruction, which emphasize authentic problem-solving, activation of prior knowledge, demonstration, application, and integration of learning, provided the theoretical basis for designing the model sessions. Guided by these principles, the study sought to develop educationally sound, interactive sessions that foster behavioral and attitudinal changes toward patient safety. To address these dual objectives, the study employed two complementary conceptual frameworks. First, to ensure systematic validation of the session plans, the Context-Input-Process-Product (CIPP) model^10^ was adopted as the conceptual framework. The CIPP model is a well-established approach to educational program evaluation that has been systematically applied across diverse contexts in higher education, including health professions education.^11^ The model examines four critical dimensions: Context (needs and relevance), Input (resources and strategies), Process (implementation quality), and Product (outcomes and effectiveness). This comprehensive structure makes CIPP particularly suited for evaluating newly developed educational interventions prior to large-scale implementation. Second, to systematically identify and categorize implementation barriers, Steinert's framework for analyzing problems in educational practice^12^ was employed, which distinguishes challenges across knowledge, attitude, skills, teacher, student, and system domains. This dual-framework approach enabled comprehensive assessment of both educational quality and implementation feasibility.

Accordingly, the present study had two interrelated aims: first, to develop and validate model session plans for patient safety learning that are educationally sound and feasible for undergraduate medical education in Japan; and second, to identify anticipated barriers to their implementation in real educational settings.

## Methods

### Study design

This study adopted a convergent mixed-methods design embedded within a modified Delphi approach to develop and validate model session plans for patient safety learning in undergraduate medical education. Quantitative and qualitative data were collected and analyzed in parallel to gain a comprehensive understanding of the sessions’ educational validity (as defined in the Introduction), feasibility, and implementation challenges. The Delphi technique was selected because it allows for the systematic collection and aggregation of expert opinions through iterative, anonymous feedback, ensuring transparency and consensus in curriculum develop-ment.^13^^,^^14^ The Delphi process was conducted in accordance with the reporting standards developed in Conducting and REporting of DElphi Studies (CREDES).^15^

### Setting and participants

Participants were recruited through purposive sampling from health professions (physicians, dentists, nurses, and pharmacists) in national university hospitals across Japan. Inclusion criteria were: (1) current employment as a certified patient safety specialist (general risk manager) or faculty member engaged in undergraduate medical education; (2) minimum of two years' experience in patient safety management or medical education; and (3) direct involvement in curriculum planning or safety training activities. Exclusion criteria included conflicts of interest such as prior involvement in developing the draft session plans or direct reporting relationships with the research team. Prior to participation, all potential panelists completed a conflict-of-interest disclosure form, and no exclusions were necessary based on these assessments.

Sample size was determined based on recommendations for Delphi studies in medical education, which suggest a minimum of 30 participants to achieve stable consensus estimates.^16^^,^^17^ Anticipating a potential 20% non-response rate, we invited 45 experts to ensure adequate panel size.

As this educational study involved minimal risk and did not include patients or personal health data, it was exempt from formal ethical review by the Ethics Committee of Chiba University Hospital. All potential participants received written information describing the study purpose, voluntary participation, and data handling procedures. Submission of the completed survey was considered informed consent. Participants were informed of their right to withdraw at any time without academic or professional disadvantage. Responses were collected anonymously through an online survey system, and data were stored on password-protected institutional servers accessible only to the research team. No financial compensation was provided.

The Delphi process was conducted in October 2024 using an online survey system. Each participant received four session plans designed for undergraduate patient safety learning and an accompanying evaluation instrument based on the CIPP model.^10^^, ^^18^

The Delphi process began with an initial round of rating and feedback, with additional rounds planned if items failed to meet the predefined consensus criteria. In this initial round, participants evaluated each of the four session plans using a 14-item CIPP-based checklist on a 4-point Likert scale ranging from 1 (strongly disagree) to 4 (strongly agree), while also providing open-ended comments regarding potential improvements or implementation challenges. The consensus definition followed established recommendations, requiring a mean score (M) ≥ 3.5 and a standard deviation (SD) < 1.0 for each item.^15^ Although Likert scale data are ordinal, the use of means and standard deviations for consensus determination in Delphi studies is widely accepted when scales have four or more points and items are assumed to reflect underlying continuous constructs.^15^^,^^17^ This approach has been validated in previous educational Delphi research and facilitates interpretation by providing intuitive thresholds for agreement levels. The predetermined stopping rule specified that additional rounds would be conducted only if items failed to meet these consensus criteria. In such cases, summary statistics and anonymized comments would be shared with the panel for subsequent review. However, because all session plans reached consensus in the first round, further iterations were unnecessary.

To ensure impartiality, participants’ responses were collected anonymously, and statistical feedback was presented without attribution. Researchers did not participate in scoring or discussion during the Delphi process. Data collection and materials. The Delphi instrument incorporated 14 evaluation criteria derived from the four dimensions of the CIPP model adapted from Stufflebeam’s checklist (Table 1).^18^ These criteria assessed (1) the relevance of learning goals, (2) appropriateness of resources, (3) effectiveness of implementation, and (4) validity of expected learning outcomes. The items were developed through a stepwise process including literature review, construct definition, item formulation, and pilot validation.

**Table 1:** Evaluation items based on the CIPP model

Domain	Item	Description
Context	CO1	The session addresses key patient safety topics
	CO2	Learning objectives are clearly defined
	CO3	The session reflects clinical and educational needs
	CO4	Learning objectives reflect patient safety needs
Input	IN1	Content is appropriate and up to date
	IN2	Feasible with available resources
	IN3	Facilitator and learner roles are clear
	IN4	Can be implemented with existing infrastructure
Process	PC1	Logical instructional flow
	PC2	Use of appropriate active learning methods
	PC3	Encourages collaborative learning
Product	PD1	Facilitates achievement of learning goals
	PD2	Assessment aligns with learning outcomes
	PD3	Expected to influence attitudes and behaviors

CO: Context items; IN: Input items; PC: Process items; PD: Product items

The draft session plans are summarized as follows. The first session, "Understanding Human Error through Everyday Failures," addressed the domain of Improvement of Healthcare Quality.² This session used familiar, non-medical failure scenarios to illustrate the universality of error and promote awareness of systemic factors. It was designed to introduce safety science concepts to early year students who may lack clinical knowledge by encouraging them to reflect on their everyday errors and reframe them using systems thinking. The goal was to build foundational awareness and connect personal experiences with improvement-oriented mindsets.

The second session, "Practicing Incident Reporting Based on Case Videos," focused on the domain of Consideration and Promotion of Patient Safety.² In this session, learners analyzed video scenarios and practiced structuring and writing effective incident reports. This paper-based simulation helped develop basic skills in incident documentation, which is a core component of a reporting culture. Using realistic video cases, the session facilitated awareness of safety concerns and fostered reflective reporting and accountability.

The third session, "Root Cause Analysis Using Simulated Clinical Cases," belonged to the domain of Safety Management System.² This exercise aimed to cultivate analytical thinking and collaborative discussion through Root Cause Analysis (RCA). By working in teams on simulated clinical scenarios, students learned how to identify contributory factors and propose preventive measures. The session served as an introductory experience to system-based error analysis within a structured safety management framework.

**Figure 1:**
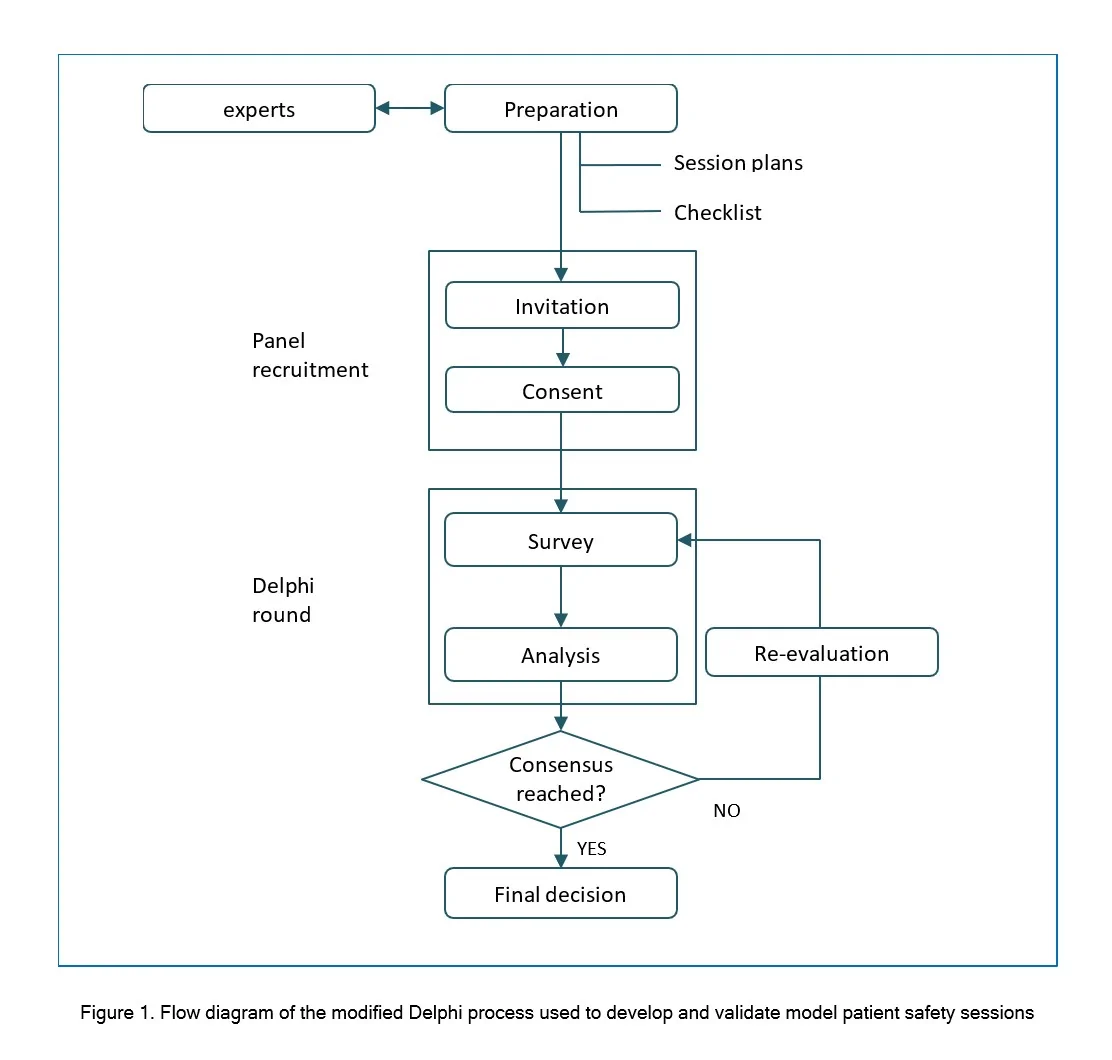
Figure 1. Flow diagram of the modified Delphi process used to develop and validate model patient safety sessions

The fourth session, "Conflict Management through Role-playing with Simulated Patients," addressed the domain of Practice of Patient Safety. ² In this session, learners developed emotional communication skills and consensus-building techniques through simulated dialogue with patients and families. The session emphasized respectful engagement with patient concerns and fostering behaviors that support psychological safety and trust. It provided experiential practice for addressing conflict situations in a constructive and patient-centered manner.

### Data analysis

The evaluation was conducted using an online questionnaire distributed to the participants through a council of general risk managers. The questionnaire included four model session plans, an instrument with 14 evaluation criteria based on the CIPP model, and a response format. Participants carefully reviewed the session plans and responded to each item on a four-point Likert scale, providing additional suggestions in the open-ended section.

### Quantitative analysis

To address the first aim of the study, descriptive statistics (mean and standard deviation) were calculated for each evaluation item. Session validity and feasibility were judged according to the predefined consensus criteria (M ≥ 3.5 and SD < 1.0). Analyses were performed using SPSS 29 (IBM).

While we recognize that reliability indices such as Cronbach’s alpha are commonly used to evaluate internal consistency in psychometric instruments, they are not conceptually applicable to Delphi studies. The Delphi technique aims to aggregate diverse expert judgments and reach consensus through iterative feedback rather than to measure homogeneous constructs. Therefore, internal consistency was not calculated, as the analytical focus was on consensus level and response stability, consistent with established Delphi methodology guidelines.^13^^-^^15^

### Qualitative analysis

To address the second aim—identifying anticipated challenges to implementing the session plans—qualitative content analysis was applied to the free-text responses collected during the Delphi process.^19^^,^^20^ Comments specifically referring to potential concerns or obstacles in implementing the proposed sessions were extracted. Data from open-ended comments were analyzed using a directed content analysis approach.^21^ This method allows for the validation and conceptual extension of a theoretical framework. We used Steinert’s framework as the initial coding scheme. The unit of analysis was defined as distinct meaning units identifying barriers to implementation. Table 2 shows the elements of the Steinert's framework and their operational definitions created through the data analysis.

### Coding and Triangulation Process

The first author (IS) categorized all data according to Steinert’s framework into the domains of knowledge, attitudes, skills, teacher, student, and system (Table 2), and refined the descriptions for each theme. Using qualitative directed content analysis, the framework served as the analytical reference.

Using an inductive approach, subthemes were generated from the data and integrated into the existing themes. The analysis proceeded through an iterative cycle of constant comparison among the data, subcategories, and categories.

The categorization and subthemes (subcategories) were then reviewed by the second author (MM) to assess their relevance and clarity within each theme. Through discussion and consensus among the authors, the subtheme labels were refined and finalized.

### Reflexivity

The analysis team consisted of educational researchers with experience in qualitative analysis (IS, MM) and a patient safety specialist (KT). We acknowledged that our involvement in the program might introduce bias. To mitigate this, we practiced reflexivity by regularly discussing our assumptions during the coding pro-cess. Furthermore, the final categorization was reviewed by the third researcher (KT), who was not involved in the direct data collection, to ensure the interpretations were grounded solely in the participants' descriptions.

### Integration of data

Following the principles of mixed-methods convergence, quantitative and qualitative findings were integrated in a joint display, allowing comparison between the validated session components (CIPP results) and the identified implementation barriers. This approach supported meta-inference on the feasibility and educational soundness of the model sessions. The learning sessions were developed through discussions among all authors, who are clinicians and researchers in medical education and patient safety, including physicians, a psychologist, and a nurse. Curriculum development and clinical relevance were discussed collaboratively. In addition to using the Delphi method to confirm the relevance of the sessions for patient safety education, qualitative directed content analysis was conducted to examine practical considerations for session implementation raised by the panelists. Drawing on their experience in undergraduate education, IS and MM identified potential instructional challenges, while KT evaluated the validity of the categorizations and the appropriateness of selected data examples.

## Results

Of the 45 invited participants, 36 completed the evaluation (response rate: 80%), exceeding the minimum threshold and ensuring robust consensus findings. The panel comprised 14 physicians, 2 dentists, 14 nurses, and 6 pharmacists.

**Table 2:** Elements of the Steinert's framework^12^ and their operational definitions created through the data analysis

Elements	Operational Definition	Inclusion Criteria	Exclusion Criteria
Knowledge	Concerns about factual understanding orinformation gaps necessary for patient safety.	Comments referencing lack of knowledge, unfamiliarity with concepts, or need forfurther learning.	General learning difficulties not attributed to knowledge deficits.
Attitude	Concerns about beliefs, values, motivation, or perceptions regarding patient safetyeducation.	Comments referencing skepticism,resistance, uncertainty about value, or role identity conflicts.	Knowledge gaps presented as factual deficits.
Skills	Concerns about technical abilities orpsychomotor capabilities.	Comments referencing inability to perform specific technical procedures.	Teaching skills (coded to Teacher); cognitive abilities (coded to Knowledge).
Teacher	Concerns about instructor preparation,teaching competence, or facilitation skills.	Comments referencing faculty experience, facilitation skills, confidence, or training needs.	System-level staffing issues (coded toSystem).
Student	Concerns about learner characteristics,readiness, or engagement patterns.	Comments referencing student preparation, prior experience with methods (e.g., peerreview), or psychological safety.	Student knowledge/attitude deficits (coded to those themes).
System	Concerns about organizational structures,resources, policies, or institutionalconstraints.	Comments referencing staffing, timeallocation, curriculum space, assessmentrequirements, or admin support.	Individual educator capabilities (coded to Teacher).

Twenty respondents held nationally recognized certifications as general risk managers, reflecting dual expertise in education and safety administration. Among educational specialists (n=16), nine held certificates or master's degrees in health professions education.

### Quantitative analysis (modified Delphi round)

All session plans met the consensus criteria (M ≥ 3.5 and SD < 1) across all 14 evaluation items based on the CIPP model (Table 3). Therefore, all plans were deemed valid and feasible in the initial round and further Delphi rounds were not required. The final session plans incorporated minor revisions and additional teaching notes based on the qualitative comments.

Table 4 shows an overview of the sessions (see Appendix). The first session, on understanding human error, was designed for preclinical students and focused on cultivating reflective thinking and system-based awareness. It used relatable examples from everyday life to engage early learners in identifying and analyzing human errors, thereby fostering vigilance and self-regulation. The second session on incident reporting targeted students transitioning into clinical training, offering foundational skills for documenting adverse events and promoting a culture of openness. The third session, which introduced RCA, was tailored for students in clinical clerkships, encouraging analytical reasoning, teamwork, and systems improvement through hands-on simulations. The fourth session on conflict management focused on learning difficult patient encounters, aiming to build empathy, emotional regulation, and mediation skills through structured role-play.

**Table 3:** Results of Delphi round

Item	Session 1: Human Error	Session 2:IncidentReporting	Session 3:RCA	Session 4:ConflictManagement
CO1	3.65 (0.68)	3.95 (0.22)	3.88 (0.40)	3.94 (0.24)
CO2	3.58 (0.69)	3.85 (0.43)	3.76 (0.54)	3.88 (0.42)
CO3	3.51 (0.67)	3.88 (0.40)	3.90 (0.38)	3.91 (0.29)
CO4	3.59 (0.73)	3.90 (0.37)	3.93 (0.27)	3.79 (0.64)
IN1	3.72 (0.73)	3.85 (0.48)	3.71 (0.52)	3.65 (0.69)
IN2	3.66 (0.75)	3.73 (0.55)	3.85 (0.43)	3.76 (0.55)
IN3	3.74 (0.69)	3.80 (0.46)	3.74 (0.45)	3.73 (0.52)
IN4	3.67 (0.74)	3.85 (0.43)	3.81 (0.46)	3.81 (0.64)
PC1	3.54 (0.78)	3.74 (0.55)	3.82 (0.51)	3.78 (0.49)
PC2	3.62 (0.72)	3.75 (0.54)	3.88 (0.40)	3.85 (0.44)
PC3	3.80 (0.68)	3.85 (0.43)	3.87 (0.41)	3.91 (0.29)
PD1	3.70 (0.63)	3.83 (0.45)	3.88 (0.33)	3.71 (0.72)
PD2	3.58 (0.84)	3.73 (0.55)	3.79 (0.52)	3.79 (0.48)
PD3	3.70 (0.78)	3.66 (0.63)	3.79 (0.53)	3.82 (0.46)

Values are presented as mean (SD). CO, Context items; IN, Input items; PC, Process items; PD, Product items; RCA, root cause analysis

#### Qualitative analysis

Of the 45 respondents, 32 provided at least one free-text comment. A total of 69 comments were collected from which 69 meaningful units were extracted for analysis. A directed content analysis approach was used, based on Steinert’s framework of six categories for barriers to educational practice: knowledge, attitude, skills, teacher, student, and system.^22^ While the six main categories were deductive, specific sub-themes were allowed to emerge inductively from the data to capture context-specific barriers.

**Table 4:** Overview of learning sessions

Session Title	Target Competency	Target Learners	Key Activity	Assessment
Understanding Human Error through Everyday Failures	Reflective thinkingHuman factors awarenessSystems thinking	Pre-clinical students	Reflective writing and pair/group discussion on non-medicalfailure experiences	Formative: peer comments and group discussion notesSummative: participation check andpost-session reflection
Practicing IncidentReporting Based on Case Videos	Incident identificationReporting cultureUnderstanding reporting systems	Pre-clinical to earlyclinical students	Writing reports based on video cases and analyzing incident scenarios	Formative: peer feedback duringpresentationsSummative: teacher observation and post-session questionnaire
Root Cause Analysis Using SimulatedClinical Cases	Analytical reasoningTeamworkQuality improvementattitude	Clinical clerkship students	Root cause analysis using simplified frameworks and development of prevention strategies	Formative: facilitator feedback onreasoning processSummative: group report assessed with rubric
Conflict Management through Role-Playing with Simulated Patients	EmpathyConflict resolutionMediationVerbal and non-verbalcommunication	Students in clinical orpre-clinical yearsapproaching patient-facing roles	Structured role-play involving emotionally charged clinical scenarios	Formative: instructor feedback during role-play Summative: group self-assessment and individual reflective report

Thirteen subthemes were identified and categorized under five of the six categories; no responses were classified under skills. The distribution of comments and representative quotes are detailed in Table 5.

Under the Teacher theme, two major subthemes emerged: insufficient competence in patient safety education (n=15) and disconnection between daily duties and academic insight (n=10). Respondents noted that teachers in patient safety departments were often unfamiliar with facilitation skills and felt that educational roles were disconnected from their primary clinical responsibilities.

The Student included subthemes related to unfamiliarity with group learning (n=2) and concerns about psychological safety (n=2). Respondents highlighted that students had limited exposure to peer evaluation or discussing failures, leading to hesitation in sharing personal errors.

The Knowledge (n=4) captured concerns regarding variation in prior knowledge, indicating uncertainty about students' baseline ability to analyze cases. In the Attitude, subthemes included variation in learner readiness (n=3) and uncertainty about specific content relevance.

The System comprised the largest number of logistical barriers, including shortage of teachers (n=11), need to engage external lecturers (n=2), misalignment with formal assessment requirements (n=4), and difficulty securing curricular slots (n=2).

#### Integrated summary

Combining the quantitative and qualitative findings, all four model sessions were validated as feasible and educationally robust within a single Delphi round. However, qualitative analysis revealed systemic issues—particularly limited teaching capacity, curricular misalignment, and institutional constraints—that could affect long-term sustainability. Table 6 presents a joint display integrating the CIPP-based quantitative validation results with the qualitative implementation barriers identified through Steinert's framework. This integration reveals patterns of convergence, divergence, expansion, and complementarity across the four CIPP dimensions. The convergence of findings suggests that faculty development and curriculum integration strategies are critical for successful implementation of patient safety learning sessions.

Accordingly, these meta-inferences directly informed the final refinements of the session plans. Specific revisions included the addition of role definitions for administrators (Context), reinforced introductory materials for novices (Input), comprehensive teaching scripts to support facilitation (Process), and streamlined assessment guidelines (Product). These modifications aim to bridge the gap between educational content quality and organizational implementation capacity.

## Discussion

Through the development and expert validation of four model session plans, this study demonstrated their educational validity and practical feasibility. As illustrated in the joint display, the integration of quantitative and qualitative findings revealed that the learning sessions are relevant to patient safety education but accompanied by structural obstacles and required implementation actions. These sessions, which emphasized experiential and collaborative learning, were aligned with expert perceptions and deemed suitable for implementation in educational settings. Active student engagement through role-play, simulations, and group discussions was particularly effective in fostering practical understanding and attitudinal shifts regarding patient safety. While the 2011 WHO Patient Safety Curriculum Guide recommended exploratory approaches such as problem-based learning, it did not provide detailed instructional strategies for each individual competency.^5^ This lack of guidance was later acknowledged by the WHO, which pointed out the need for more specific and practical educational approaches to support implementation.^4^ The present study addressed this gap by providing structured, theory-informed, and contextually adaptable session plans that concretize the WHO framework into reproducible teaching models. These findings extend previous research on patient safety curricula by demonstrating that practical, competency-linked sessions can be both educationally sound and feasible for nationwide implementation.^23^^,^^24^ Although the models achieved strong consensus among panelists, practical considerations for implementation remain. Addressing these considerations and disseminating them to educational institutions and faculty may facilitate effective adoption of the sessions.

The session plans developed in this study were designed not merely for knowledge transmission but to promote behavioural change and ethical reflection. They also provided students—particularly those unfamiliar with peer learning or reflective practices—with structured exposure to key competencies rarely encountered in traditional curricula.” Activities such as sharing failure experiences and simulating conflict management were effective in reducing psychological barriers to incident reporting and fostering non-technical skills such as empathy and self-reflection.^25^ These practices contributed to the cultivation of a safety culture and the enhancement of ethical medical behavior. Furthermore, as shared educational resources, the sessions can be adapted to the contextual needs of individual institutions and may contribute to elevating the national standards of patient safety education.^4^ By aligning each session with the Model Core Curriculum and the principles of competency-based medical education, the design ensured coherence between intended learning outcomes, instructional processes, and assessment strategies—an approach consistent with current global standards in medical education.

**Table 5:** Reported barriers to implementing patient safety learning sessions in this study

Themes	Subthemes	Number	Example quotes (translated)
1. Knowledge	Consideration of appropriate sequence of learning	5	“Students may find it easier to develop recurrence prevention strategies after first learning basic preventive measures.”
	Variation in prior knowledge	1	“It’s unclear how well students can analyse and plan countermeasures for the cases.”
2. Attitude	Unfamiliarity with group learning	5	“My students lack experience with peer assessment, raising concerns aboutfree-riding and cheating.”
	Variation in learner readiness	2	“I’m unsure whether learners are ready to perform root cause analysis onreal-world issues.”
3. Student	Concerns about psychological safety	2	“Students may hesitate to disclose their own mistakes.” “It may be necessary to practise pair work with guidance to avoid blaming oneanother for failures.”
4. Teacher	Insufficient competence in patient safety education	15	“Instructors themselves must be familiar with these methods.” “Educators are unfamiliar with syllabus development.”
	Disconnection between daily duties and academic insight	10	“As a patient safety expert, I struggle to see connections with everyday work.” “I perceive conflict management as separate from patient safety.”
	Context-specific adjustments	7	“I'm concerned that the progression may vary depending on faculty availability.” “It may be acceptable to deliver the lecture and the role-play in separate sessions.”
5. System	Shortage of teachers	11	“It may be difficult to secure enough facilitators.” “Securing time, space, and responsible staff for instruction is challenging.”
	Misalignment with formal assessment requirements	4	“There are too many points to assess; I question whether proper evaluation ispossible.” “If only parts of the activity are implemented, I need confirmation that it still meets the curricular grading requirements.”
	Effort management challenges	3	“Educational duties are not usually included in the job description of full-timepatient safety officers.”
	Need to engage external lecturers	2	“In universities without affiliated hospitals, it may be necessary to invite external professionals from safety offices.”
	Difficulty securing curricular slots	2	“It’s unclear whether time can be allocated within the curriculum.”

Note: No responses were classified under skills

**Table 6:** Joint Display integrating quantitative CIPP evaluation results with qualitative implementation barriers based on Steinert’s framework

CIPP	Items	QuantitativeFindings	Qualitative Findings		Mixed Methods Interpretation and Action
		Mean Range	Key Barriers	Themes	
Context	CO 1–4	3.51–3.91	- Effort management challenges	System	While the quantitative results confirmed that the session objectives align with curriculum goals, qualitative themes revealed a systemic gap in organizational clarity (e.g., job descriptions, dual roles of safety managers). This indicates that the barrier is not the content itself, but the lack offormal recognition of educational duties.(Action) The final implementation guide was revised toinclude clear role definitions and workload allocationmodels to support administrators in legitimizing theseeducational activities.
			- Disconnection between daily duties and academic insight	Teacher	
Input	IN1–4	3.66–3.76	- Shortage of teachers	System	Although materials were rated as sufficient quantitatively, qualitative data exposed critical resource constraints—specifically, a shortage of trained facilitators and variability in students' prior knowledge. This suggests that "content readiness" does not equal "implementation readiness."(Action) The final package was reinforced with introductory modules for students (to level baseline knowledge) and recruitment strategies for external lecturers to address staffing shortages.
			- Need to engage external lecturers	System	
			- Difficulty securing curricular slots	System	
			- Insufficient competence in patient safety education	Teacher	
			- Variation in prior knowledge	Knowledge	
			- Variation in learner readiness	Attitude	
			- Unfamiliarity with group learning	Attitude	
Process	PC 1–3	3.74–3.82	- Insufficient competence in patient safety education	Teacher	High feasibility scores for instructional methods were nuanced by qualitative concerns regarding psychological safety and facilitation difficulty (e.g., managing group dynamics, peer review). This implies that even well-designed processes can fail without skilled execution.(Action) Comprehensive teaching scripts and frequently asked questions were embedded into the instructor manual to scaffold non-expert facilitators and ensure a safe learning environment.
			- Context-specific adjustments	Teacher	
			- Concerns about psychological safety	Student	
			- Psychological safety concerns	Student	
			- Consideration of appropriate- sequence of learning	Knowledge	
Product	PD1–3	3.58–3.72	- Misalignment with formal assessment requirements	System	Quantitative ratings affirmed that learning outcomes are measurable, but qualitative feedback highlighted the administrative burden of assessment (e.g., grading requirements). This reveals a tension between "ideal assessment" and "practical feasibility." (Action) Streamlined assessment guidelines aligned with institutional grading policies were added, offering optional simplified formats to reduce faculty workload while maintaining academic rigor.

All quantitative findings met consensus criteria. Note: Some subthemes, such as 'Difficulty securing curricular slots', have implications for both Input and Context dimensions but are categorized here based on their primary impact.

To enhance content validity, a modified Delphi method involving experts from multiple disciplines was employed during the development of the session plans.^26^ For instance, the session on human error is well-suited for early-stage learners as part of foundational training in ethics or professionalism, while the sessions on incident reporting and conflict management are appropriate for integration into preclinical curricula. These sessions are also adaptable for post-graduate education, enabling residents to reinforce their non-technical patient safety competencies. The inclusion of multi-professional experts in the Delphi panel enhanced the credibility and transferability of the findings across diverse educational settings. Notably, insufficient teaching competence and shortage of teachers were the most frequently cited barriers, underscoring the critical need for systematic faculty development initiatives. Given that many general risk managers lack experience in curriculum design or active learning facilitation, targeted faculty development should address not only content knowledge but also instructional strategies.^27^ Developing structured faculty development programs that focus on facilitation, feedback, and assessment techniques could further support effective dissemination of these model sessions. ^28^

All four session plans were designed for class-room-based delivery and did not include workplace-based learning. This was for two main reasons. First, the timing and structure of workplace-based learning vary significantly across specialties, making standardization difficult.^29^ Second, current curricula offer limited practical learning opportunities specifically focused on patient safety.^7^^,^^30^ No barriers related to skills were identified in the qualitative analysis. This aligns with the nature of the patient safety competencies, which focused on cognitive and non-technical domains rather than technical skills used for clinical procedures. Developing patient-safety-oriented clinical rotations remains an important future direction. Future research should explore how these classroom-based sessions can be integrated with simulation-based and work-place-based learning to create a longitudinal learning continuum that strengthens behavioral outcomes in real clinical environments.

Moreover, a key feature of the proposed sessions is the integration of structured learner assessment strategies. In competency-based education, it is essential to assess whether learners can apply acquired skills in real-world practice.^31^ However, assessment of educational effectiveness remains a significant weakness in existing patient safety education.^28^ By embedding assessment strategies into each session, this model addresses that gap and strengthens both the accountability and practical utility of patient safety instruction. Such embedded assessments can also facilitate program evaluation aligned with the CIPP framework used in this study, thereby linking instructional design and quality improvement in education.

Based on the findings from the qualitative content analysis, several practical considerations emerged regarding the implementation of these sessions in real-world educational contexts. These implementations challenges aligned with barriers identified through content analysis, particularly in the categories of teacher, student, and system. The results clarified limited instructional experience among safety educators and a lack of prior exposure among students to activities such as peer assessment or reflective discussion.^5^^,^^32^ Structural constraints—such as shortages in teaching staff, absence of formal teaching roles for safety officers, and limited curricular space—also emerged as key concerns. Addressing these multi-level barriers is essential for the sustainable integration of safety education into health professions curricula. For example, a co-facilitation model involving both clinical educators and safety officers, or the development of brief training modules for facilitators, may help overcome structural barriers.^33^ Furthermore, it is reported that job descriptions may act as barriers for education in the work-place.^34^ Aligning the educational responsibilities of general risk managers with their job descriptions could promote more sustainable integration of these sessions into routine educational practice. These findings reinforce the importance of institutional commitment and leadership support for patient safety education, suggesting that organizational culture change must accompany pedagogical innovation to achieve long-term sustainability.^35^

In some cases, these structural and educational challenges may reflect perceptions towards patient safety management practices. Traditional approaches often emphasise strict procedural compliance and focus on individual deviations. While recent directions in patient safety increasingly integrate insights from safety engineering and cognitive psychology, our findings suggest that some practices may still be rooted in classical, rule-based understandings of safety management.^36^ Some participants questioned whether conflict management fits within patient safety education. This reflects a common divide between clinical responsibilities for patient safety and the educational roles of safety departments. While patient safety offices often focus on system oversight, they also have a duty to support education that enables safer clinical practice. Teaching conflict management is a prime example. It builds communication and de-escalation skills essential for patient-centered care. By contributing to such educational efforts, patient safety staff help develop future professionals who can act safely and collaboratively in complex clinical environments. At the same time, involvement in undergraduate curriculum offers a valuable lifelong learning opportunity for general risk managers themselves. This dual benefit—educating students while simultaneously enhancing educators’ own reflective practice—illustrates how patient safety education can serve as a platform for mutual professional growth and organizational learning.

This study had several limitations. First, although the validity and feasibility of the session plan were demonstrated based on expert opinions, future studies should verify educational outcomes through practical implementation. Second, a methodological limitation is that the Delphi process concluded in the first round as all items met the strict pre-determined consensus criteria immediately. Consequently, the iterative feedback and re-evaluation loop—a hallmark of the Delphi method—was not utilized, meaning the results reflect initial expert agreement rather than opinion convergence over time. Third, because all panelists were affiliated with university hospitals, the generalizability of the findings to other medical professional education facilities (e.g., junior colleges and vocational schools) is limited. These facilities are often constrained by shorter curricula, which may limit the opportunity to introduce new opportunities for a deeper understanding of patient safety. In addition, this study did not assess long-term retention or behavioral change among learners, which represents a key area for further research. Repeating the cycle of evaluation and design improvement when implementing the sessions proposed in this study is necessary to enhance session quality. Future multi-institutional or longitudinal studies will be necessary to verify transferability and sustainability across different educational contexts. Longitudinal studies that verify behavioral outcomes can support the establishment of evidence-based patient safety learning. Despite these limitations, this study contributes to a replicable and theory-informed model that can inform future curriculum development and strengthen the foundation for patient safety education in Japan and beyond.

## Conclusions

This study developed four model session plans for undergraduate patient safety learning in Japan and verified their validity and feasibility through expert evaluations involving general risk managers and faculty members from national university hospitals. All sessions met the consensus criteria in the first evaluation round and were educationally sound. Covering key themes, namely, human error, incident reporting, RCA, and conflict management, the sessions promoted experiential and collaborative learning to support behavioral and attitudinal change. Future practical research should test the sessions’ effectiveness and impact on student behavior, along with efforts to enhance faculty facilitation skills. Longitudinal and multi-institutional evaluations are warranted to confirm the transferability, sustainability, and behavioral outcomes of these sessions in diverse educational contexts. In addition, faculty development programs that strengthen facilitation, feedback, and assessment skills are essential to maximize educational impact and ensure quality implementation. These efforts are expected to support the national adoption and institutionalization of patient safety education. In parallel, the findings from our qualitative analysis indicate that several barriers must be addressed to ensure sustainable implementation. Practical solutions to target identified barriers are expected to support the national adoption and institutionalization of patient safety education.

### Acknowledgement

We thank the panelists for their participation in the Delphi round. We thank the members of the National University Hospital Patient Safety Management Council's Committees on Health Professions Training and Undergraduate Education for their advice. We would also like to thank Editage for English language proofreading.

### Conflict of Interest

The authors declare that there is no conflict of interest.
