# Supplementary material for: Developing and validating patient safety sessions in Japanese medical education: a modified Delphi study
Source: Int J Med Educ. 2026 May 11;17:42–60. doi: 10.5116/ijme.69f0.78c6 (PMC13563279; doi:10.5116/ijme.69f0.78c6)
Supplement: Supplementary file 1 [file ijme-17-42-S1.pdf]

Appendix

Session Plan 1: Learning from Everyday Failures

1. Relevant Competencies and Learning Objectives

1.1. Model Core Curriculum for Medical Education (Japan)
- PR-01-02-01: Recognize and act within one's limitations
- PR-01-02-02: Accept feedback from others appropriately
- LL-01-02-01: Reflect on experiences and identify personal learning needs
- LL-01-02: Career Development
- LL-01-02-02: Care for one's physical and mental well-being
- LL-02: Health Professional Education
- LL-02-01: Learn collaboratively with peers and colleagues
- LL-02-01-02: Provide appropriate feedback to others
- CS-05-01: Understand and repeatedly apply quality improvement methods in healthcare

1.2. Training Guidelines for General Risk Managers

1. Basic knowledge of patient safety

6. Fostering a safety culture

1.3. Learning Objectives:
- Collaboratively reflect on one's own everyday failure and consider measures to prevent recurrence.
- Understand the significance and organizational impact of reflecting on failure, and think about the role and value of incident reporting systems.

2. Learning Strategy

2.1. Overview

This session introduces the concept of incident reporting by using everyday non-medical failure experiences as a foundation for reflection and peer discussion. Through writing and sharing, students develop foundational skills for reporting and learning from incidents in a healthcare context.

2.2. Session Activities
- Introduction Lecture (10 minutes): Concept and importance of "reflection"; example of incident reporting; instructions for session activities.
- Activity 1: Reflective Writing (15 minutes total): Reflect on and analyze a personal everyday failure experience.
- Activity 2: Pair Sharing and Analysis (15 minutes): Exchange narratives; give/receive feedback; discuss prevention strategies.
- Activity 3: Group Discussion (15 minutes): Reflect on organizational implications of sharing failures; facilitated and recorded.
- Activity 4: Class-wide Sharing (5 minutes)
- Wrap-up and Q&A (10 minutes)

2.3. Facilitators

General risk managers, faculty from the patient safety office, and educators experienced in reflection and incident reporting.

2.4. Duration

90 minutes

2.5. Setting

Lecture hall with tables for group work; tiered seating acceptable

2.6. Target Learners

Primarily pre-clinical undergraduate students; full cohort participation possible

2.7. Materials Needed

Worksheet with 'Fact – Analysis – Prevention'; collaborative presentation software or poster paper and markers for group work

3. Assessment

3.1. Goals for Achievement
- Reflect on one's personal failure and collaboratively explore recurrence prevention strategies.
- Consider the meaning of failure reflection and its relevance to the function of incident reporting systems.

3.2. Assessment methods
- Formative Assessment: Peer feedback and discussion contributions recorded by scribes; faculty feedback as needed.
- Summative Assessment (Pass/Fail): Participation essential; teacher observes group work; post-session questionnaire assessed.
- Make-up report and discussion required for absentees or non-participants.

Session Plan 2: Experiencing the Incident Reporting System

1. Relevant Competencies and Learning Objectives

1.1. Model Core Curriculum for Medical Education (2022 version):
- CS-05: Quality and Patient Safety
- CS-05-05-01: Able to implement basic preventive strategies (e.g., patient identification, double-checking, checklists, alerts for look-alike/sound-alike medications, fail-safe and foolproof principles).
- CS-05-05-02: Able to share safety-related information (e.g., adverse drug reactions, medical accidents, prohibited practices, good practices) and analyze it for future use.

1.2. Training Guidelines for General Risk Managers:
2. Building safety management systems
4. Collecting, analyzing, and feeding back safety information
6. Fostering a safety culture

1.3. Learning Objectives:
1. Understand the incident reporting system and gain practical experience using it as a user.
2. Express one's awareness and ideas within a group of health professionals (undergraduate level) and engage in mutual discussion.

2. Learning Strategy

2.1. Overview
This session allows learners to understand the structure and purpose of incident reporting systems, practice report writing, and simulate analysis and recurrence prevention strategies. Group discussions foster understanding of how such systems affect personal practice and organizational learning.

2.2 Session Activities
- Introductory Lecture:
  - ✧ Overview of the incident reporting system (origin, structure, format).
- Writing Exercise (individual work and pair feedback):
  - ✧ Based on a prepared case, learners write a report as if they had experienced the event themselves.
  - ✧ Emphasis on clarity and factual description.
- Incident Analysis (individual work and group sharing):
  - ✧ Use a safety analysis framework (e.g., foreseeability, culpability, classification as an incident).
  - ✧ Identify underlying issues (e.g., medication errors, communication breakdowns).
  - ✧ Formulate questions to clarify contributing factors.
- Recurrence Prevention Strategy (group work):
  - ✧ Select one key issue and propose a prevention strategy.
  - ✧ Consider what organizational information must be clarified to design effective measures.
- Group Discussion:
  - ✧ Discuss the impact of incident reporting on organizations and individuals.
  - ✧ Sample discussion questions:
  - ✧ How does having or lacking a reporting system affect an organization?
  - ✧ Should systems vary by facility size?
  - ✧ How can we close the PDCA cycle with feedback, and what barriers exist?
- Peer Presentations:
  - ✧ Groups present findings and exchange assessments using a rubric.

2.3. Facilitators
General risk managers, faculty from the patient safety office, and instructors knowledgeable about incident reporting systems.

2.4. Duration
90 minutes for lecture and analysis work; 180 minutes for the full session.

2.5. Setting
Large lecture hall; flat tables preferred but tiered seating acceptable.

2.6. Target Learners
All students in a cohort (as preparatory education or during clinical training).

2.7. Curriculum evaluation
Student surveys: understanding and reflections; tracking how many students submit reports during clinical training.

2.8. Required Resources

Real or simulated incident cases and reporting forms; collaborative presentation software or poster paper and pens for group work.
2.9. Assessment
- Attendance and participation in group work required.
- Summative assessment via faculty observation and written reflections.
- Formative assessment includes peer feedback during presentations.

Rubric Example

Rating	Excellent	Good	Pass	Fail
Description	Demonstrates deep reflection on the session content, links to professional responsibilities.	Shows some reflection and understanding of the session.	Understands content but lacks significant reflection.	Lacks comprehension or shows unsafe beliefs; disregards format or word count.
Criteria	Excellent	Good	Satisfactory	Unsatisfactory
Respect for Patient's Wishes	Fully respected and clearly understood patient wishes	Respected, but partially unclear	Insufficient understanding	Ignored patient wishes
Sensitivity to Family Emotions	Clearly understood and empathized	Some empathy shown	Attempted but inadequate	Lacked or disrupted empathy
Dialogue Support and Options	Achieved all: asked both sides, supported dialogue, adjusted properly	Achieved two	Achieved one	No intention to mediate
Clarity of Communication	Clear, concise, complex info well explained	Mostly clear, some unclear parts	Basic info, partly unclear	Unclear and confusing
Non-Verbal Communication	Effective eye contact, tone, gestures	Positive non-verbal signals	Limited effect	Poor impression
Emotional Control	Calm under stress	Generally controlled	Sometimes emotional	Poor control

Session Plan 3: Root Cause Analysis (RCA) of Medical Incidents

1. Relevant Competencies and Learning Objectives

1.1. Model Core Curriculum for Medical Education (2022 version):
- CS-05-05-01: Able to implement basic preventive measures (e.g., patient identification, double-checking, checklists, alerts for look-alike/sound-alike medications, fail-safe/foolproof concepts).
- CS-05-05-02: Able to share safety-related information (e.g., adverse reactions, falls, malpractice, forbidden acts, best practices) and analyze such events for future improvement.

1.2. General Risk Manager Training Guidelines:

4. Collection and analysis of safety data, feedback, and countermeasure planning
6. Cultivating a safety culture

1.3. Learning Objectives:
- Simulate and practice the Root Cause Analysis (RCA) method for medical incidents.
- Formulate recurrence prevention strategies.

2. Learning Strategy

2.1. Overview

This session is designed for clinical rotation periods, focusing on simulated exercises of RCA and the development of recurrence prevention strategies. The format assumes a rotation through the patient safety office but may be coordinated with clinical departments or wards.

RCA is used to identify what happened, why it happened, and how to prevent recurrence. The emphasis is on exploring "how" and "why," rather than "who" is to blame.

2.2. Session Activities
- Pre-lecture (20 minutes):
  - ✧ Delivered via on-demand video. Completion of a brief quiz is required for participation.
  - ✧ Topics: organizational response to incidents, incident reporting systems, contributing factors to accidents, steps for root cause analysis.
- Case Analysis (2 hours):
- Present incident cases using paper or slides (real or famous, with prior analysis and clear relevance).
  - ✧ Encourage understanding through visual aids or reenactment videos.
  - ✧ Conduct analysis using simplified frameworks like the London Protocol or RCA.
- Development of Recurrence Prevention Strategies (1 hour):
  - ✧ Based on the most relevant and addressable factor(s), propose actionable strategies.
  - ✧ Define "who will do what by when."
  - ✧ Emphasize institutional or procedural solutions over individual responsibility.
- Debriefing and Feedback Session (15 minutes):
  - ✧ Explain what analysis and strategies were implemented in the actual case.
  - ✧ Allow for Q&A

2.3. Example of Analysis Method (Simplified London Protocol)
- ✧ Timeline Chart: Organize events by person (columns) and time (rows).
- ✧ Brainstorming Contributing Factors: Each student writes at least 5 factors on sticky notes and posts them.
- ✧ Categorization and Mapping: Sort notes by theme and map relationships with arrows.
- ✧ 5 Whys Technique (Condensed): Explore root causes up to 3 times for time efficiency.
- ✧ Strategy Development: Specify concrete actions ("who," "by when," "what").
- ✧ Comparison: Present actual RCA findings and countermeasures.

2.4. Facilitators
- ✧ General risk managers, faculty in the patient safety office, and clinicians involved in the case (as needed).

2.5. Duration

Half-day session; optional 15-minute pre-learning material.

2.6. Setting

Small-group room during clinical safety rotations; larger rooms or online delivery possible.

2.7. Learners

Students in clinical training; typical group size: 6–8 students; also suitable for large workshops.

2.8. Curriculum evaluation

Student questionnaire; patient safety attitude scale.

2.9. Required Resources

Incident cases and actual incident reports; sticky notes (20 per person), writing materials; large paper sheets or whiteboard.

2.10. Assessment

- Formative Assessment: Conducted during the exercise by the facilitator, based on analysis depth, use of reasoning, and strategy feasibility. Comments are recorded.
- Summative Assessment: Final report assessed using a rubric by the rotation supervisor.

Rubric Example

Criteria	Excellent	Good	Fair	Poor
Participation	Actively offers constructive suggestions for analysis and prevention strategies	Contributes relevant suggestions	Offers general comments only	Does not contribute or holds problematic safety beliefs
Report	Reflects on lecture content, connects to professional responsibilities	Shows basic reflection	Limited self-reflection	Fails to engage with content or format

Session plan 4. Conflict Management through Mediation

1. Relevant Competencies and Learning Objectives

1.1. Model Core Curriculum for Medical Education (2022 version):
- CS-05-05-01: Able to implement basic preventive measures (e.g., patient identification, double-checking, check-lists, alerts for look-alike/sound-alike medications, fail-safe/foolproof concepts).
- CS-05-05-02: Able to share safety-related information (e.g., adverse reactions, falls, malpractice, forbidden acts, best practices), and analyze such events for future improvement.

1.2. General Risk Manager Training Guidelines:
- 5. Incident response
- 6. Cultivating a safety culture

1.3. Learning objectives
- Understand conflict management as an approach to effectively resolve interpersonal tension and disagreement and recognize mediation as a key technique within it.
- Demonstrate basic mediation techniques in simulated clinical or interpersonal scenarios.

2. Learning Strategy

2.1. Overview

Conflicts between healthcare providers and patients/families may arise due to differing values, goals, expectations, and perspectives. Such conflicts, especially following adverse events, can hinder proper responses. Thus, healthcare providers must understand conflict management and mediation.

While this session is ideally suited for students with some clinical knowledge, it is also understandable for those before clinical clerkship.

2.2. Session Activities

A two-period (90 minutes each) session combining lecture with multiple staged role-plays (role-play (RP)). Role-play has been shown to be effective in developing medical students' communication skills, especially listening skills.
- Introduction (10 minutes): Explain the session's flow.
- RP1: "The Fate of the Orange" (30 minutes)

    - Goal: Understand interpersonal conflict and how it can be resolved through dialogue.
    - Content: Students form pairs and role-play as siblings fighting over a single orange. With only the scenario explained, they must choose one of the following actions: A) Give it to the sister B) Give it to the brother C) Split it in half D) Engage in mutual dialogue
    - After the RP, the instructor discusses:
    - Conflict management involves understanding each other's interests. Through discussion, it is revealed that the sister wants to make marmalade and the brother wants to make juice.
    - They collaborate to use the peel and juice, respectively.
    - This illustrates how dialogue reveals interests and builds consensus.

- Lecture (30 minutes)
    - ✧ Goal: Understand medical conflicts and mediation as a resolution technique.
    - ✧ Define conflict, explore causes and consequences in healthcare.
    - ✧ Explain mediation's significance and procedure.
    - ✧ Conclude with 1-2 quiz questions and immediate discussion.

- RP2: Reception Conflict Due to Scheduling Error

    - Groups of six: patient, spouse, child, doctor, mediator, evaluator.
    - Goal: Simulate conflict management between a patient and staff.
    - Scenario: A patient arrives for an outpatient appointment, but the appointment isn't in the system. The patient insists they changed the date a week prior, but records are missing and no one can verify the miscommunication.
    - The patient demands to see their doctor, who is fully booked. The doctor offers to see them last, but the patient needs an earlier consultation.
    - Roles:
        - ➢ Patient: Frustrated, insists on being seen quickly
        - ➢ Receptionist: Apologetic, seeks to calm and resolve
        - ➢ Hospital Staff: Supports communication and mediates
    - Task: The hospital staff supports non-judgmental communication and explores collaborative solutions., explores patient needs and possible solutions.

- Wrap-up and Q&A

- ✧ Gather reflection summaries from selected groups.
- ✧ Share key points on consensus-building.
- ✧ Open Q&A.

2.3. Facilitators
- Staff from the Hospital Safety Office (e.g. general risk managers)
- Mediators, palliative care team members, etc.

2.4. Duration
180 minutes in total

2.5. Setting
A classroom setting is assumed; however, a layout that facilitates group work is preferable.

2.6. Target Learners
The session is primarily intended for students prior to clinical clerkships, but it can also be implemented in earlier years or during and after clerkships. Designed as a workshop delivered to an entire cohort.

2.7. Curriculum Evaluation
- Student feedback questionnaires
- Measures assessing patient safety awareness

2.8. Required Resources
Printed role-play scenarios
Group assignments, prearranged group allocation

3. Assessment
- Formative: Conducted during RP by instructors. Feedback focuses on product completeness, reasoning depth, and effectiveness of solutions. Notes are taken for reference in summative assessment.
- Summative: Students self-evaluate RP2 using a rubric and submit a summary. Individual written reflections are collected.

Sample rubric for role-play Assessment

A. Role-play performance

Criteria	Excellent	Good	Fair	Poor
Respect for patient preferences	Fully respected and clearly understood the patient's preferences	Respected patient preferences but some aspects were not clearly communicated	Insufficient understanding of patient preferences	Ignored patient preferences
Consideration of family emotions	Accurately understood family emotions and responded empathetically	Showed consideration for family emotions but empathy was insufficiently expressed	Attempted to address family emotions but responses were inadequate	Failed to consider family emotions or aggravated them
Facilitation of dialogue and generation of options	Achieved all of the following:<br>- Asked both patient and family questions and<br>- elicited perspectives<br>- Facilitated dialogue based on their views<br>- Appropriately mediated the discussion	Achieved two of the Excellent criteria	Achieved one of the Excellent criteria	No intention to mediate or reconcile differing views
Clarity of communication	Provided clear and concise explanations and conveyed complex information understandably	Communicated clearly overall but some complex information remained unclear	Delivered basic information but with some lack of clarity	Explanations were often unclear and difficult to understand
Non-verbal communication	Effectively used eye contact, tone of voice, and body language	Displayed adequate non-verbal communication and conveyed a positive impression	Used non-verbal communication but with limited effectiveness	Lack of non-verbal communication resulting in a negative impression
Emotional regulation	Maintained composure and responded appropriately in stressful situations	Generally controlled emotions but occasional emotional responses were observed	Some emotional control but periodic emotional reactions occurred	Unable to regulate emotions appropriately

B. Written report

Criteria	Excellent	Good	Fair	Poor
Participation	Actively contributed constructive opinions supporting analysis and development of preventive strategies	Provided opinions related to analysis and preventive strategies	Expressed opinions but limited to general impressions	Did not express opinions or demonstrated problematic beliefs regarding patient safety
Reflection in report	Demonstrated understanding of the lecture and workshop, reflected on personal involvement and learning, and discussed professional implications for future practice	Showed understanding of the session with some reflection on learning	Understood the session but showed minimal reflection	Did not reflect the session content, held problematic patient safety beliefs, or failed to meet required format or length
